# Supplementary material for: Causal Relationship between Aging and Anorexia Nervosa: A White-Matter-Microstructure-Mediated Mendelian Randomization Analysis
Source: Biomedicines. 2024 Aug 16;12(8):1874. doi: 10.3390/biomedicines12081874 (PMC11351342; doi:10.3390/biomedicines12081874)
Supplement: Supplementary file 1 [file biomedicines-12-01874-s001.zip › Supplementary Figure/Figure S9/Leaveoneout plot of MR analysis for ubm-b-1923 to AN.pdf]

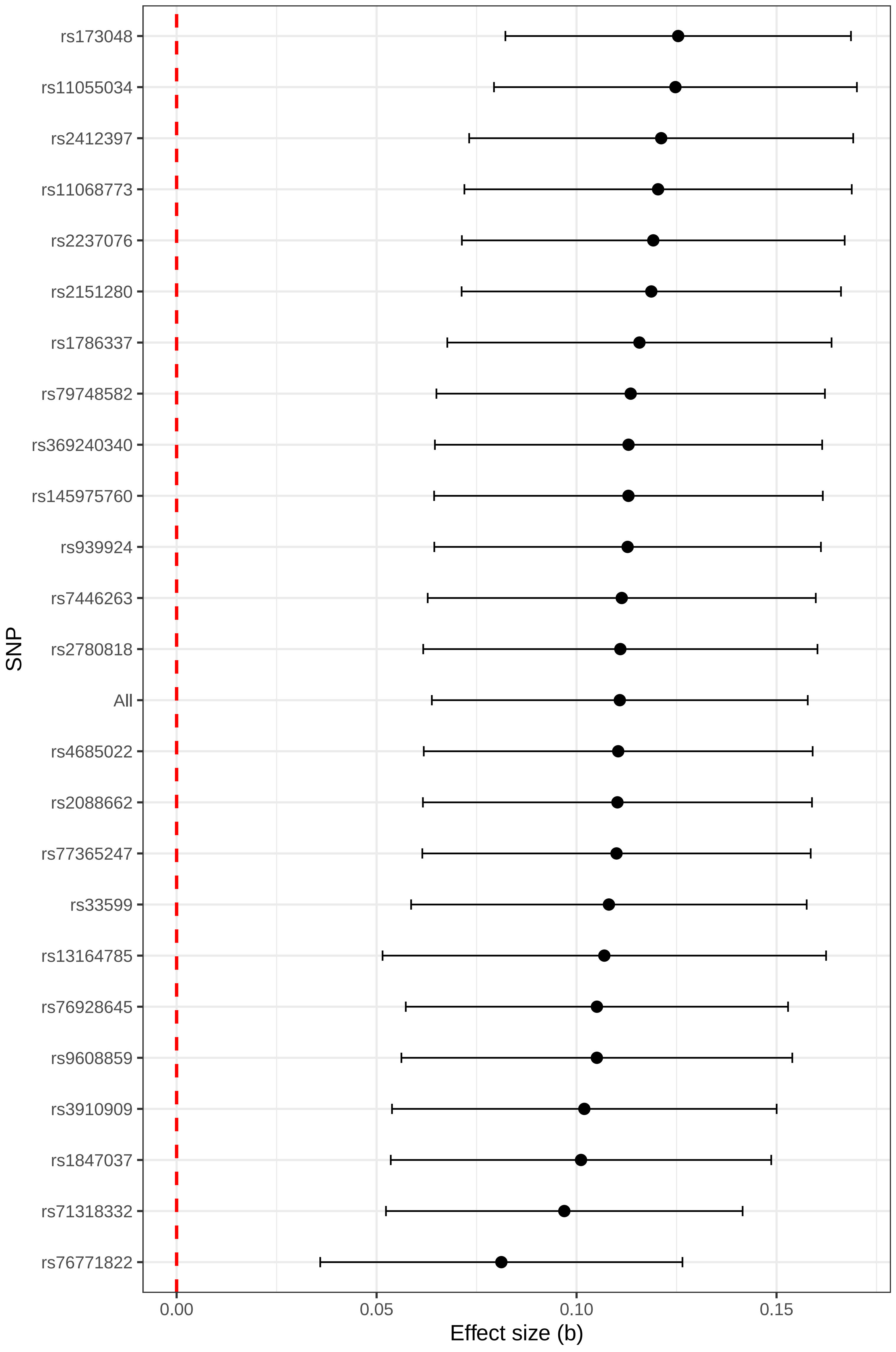

rs173048
rs11055034
rs2412397
rs11068773
rs2237076
rs2151280
rs1786337
rs79748582
rs369240340
rs145975760
rs939924
rs7446263
rs2780818
All
rs4685022
rs2088662
rs77365247
rs33599
rs13164785
rs76928645
rs9608859
rs3910909
rs1847037
rs71318332
rs76771822
SNP
0.00
0.05
0.10
0.15
Effect size (b)
